# Therapeutic strategies in prion disease: current evidence, translational challenges, and emerging directions

**DOI:** 10.3389/fnins.2026.1924347

**Published:** 2026-09-03

**Authors:** Yixin Zhu, Barry M. Bradford, Neil A. Mabbott

**Affiliations:** The Roslin Institute and Royal (Dick) School of Veterinary Studies, University of Edinburgh, Easter Bush, Midlothian, United Kingdom

**Keywords:** antisense oligonucleotides, astrocytes, Creutzfeldt–Jakob disease, microglia, prion diseases, prion protein, *PRNP*, therapy

## Abstract

**Background:**

Prion diseases are rare, transmissible, and invariably fatal neurodegenerative disorders caused by the conformational conversion of cellular prion protein (PrP^C^) into its pathogenic isoform (PrP^Sc^). Despite decades of research, no licensed disease-modifying therapies are currently available. This reflects the rapid clinical course of these disorders, the difficulty of early diagnosis, and the biological challenge of targeting a self-propagating protein misfolding process within the central nervous system.

**Results:**

This narrative scoping review maps the current therapeutic landscape in prion disease, with emphasis on the molecular mechanisms of pathogenesis, historical and emerging treatment strategies, and other exploratory therapeutic candidates. The included literature covers PrP-lowering strategies, anti-PrP immunotherapy, downstream modulation of glial dysfunction, and broader translational barriers that continue to limit clinical development. Overall, therapeutic research has shifted from repurposed small molecules toward more mechanism-based strategies. These include *PRNP*-targeting strategies using antisense oligonucleotides, siRNA, and genomic editing technologies, as well as anti-PrP immunotherapy. Collectively, these strategies provide a stronger biological rationale than earlier compounds, although evidence from human trials remains limited, early-phase, or absent. In parallel, increasing evidence indicates that maladaptive glial cell activation and chronic neuroinflammation contribute substantially to disease progression and may represent additional therapeutic targets.

**Conclusion:**

Current evidence supports increasing emphasis on integrated, mechanism-based strategies that combine suppression of prion propagation with modulation of downstream tissue injury. However, evidence of clinical efficacy in human patients is lacking. Future progress will depend on earlier diagnosis, improved translational models, and more rigorous human evaluation.

## 1 Introduction

Prion diseases, collectively referred to as transmissible spongiform encephalopathies (TSEs), are invariably fatal neurodegenerative disorders caused by the conformational conversion of the cellular prion protein (PrP^C^) into its pathogenic misfolded isoform (PrP^Sc^) (Johnson, 2005). The progressive accumulation of PrP^Sc^ within the central nervous system (CNS) drives a cascade of neuropathological events, including spongiform vacuolation, synaptic dysfunction, neuronal loss, microgliosis and reactive astrocytosis, ultimately resulting in rapid and irreversible neurodegeneration (Chen et al., 2022; Thellung et al., 2018; Lee et al., 2013).

Prion diseases occur in both humans and other mammalian species (Barria et al., 2014; Gallardo and Delgado, 2021). In animals, scrapie has long been endemic in sheep and goats, chronic wasting disease (CWD) is established in cervids, and bovine spongiform encephalopathy (BSE) demonstrated the zoonotic potential of animal prion disorders through its link to variant Creutzfeldt–Jakob disease (vCJD) in humans (Gallardo and Delgado, 2021; EFSA Panel on Biological Hazards (BIOHAZ) et al., 2019). In humans, Creutzfeldt–Jakob disease (CJD) is the most common prion disease and is classified into sporadic, familial, iatrogenic, and variant forms (Gambetti et al., 2003; Iwasaki, 2017). Sporadic CJD (sCJD) accounts for approximately 85–90% of cases worldwide and occurs at an incidence of around 1–2 cases per million population annually (Gambetti et al., 2003; Iwasaki, 2017; Uttley et al., 2020). Despite advances in diagnostic techniques and supportive care, the prognosis remains extremely poor, with median survival typically less than 1 year from symptom onset (Miranda et al., 2022). The absence of an effective disease-modifying treatment reflects one of the greatest unmet needs in contemporary neurodegenerative medicine.

The difficulty of developing therapies for prion disease reflects the complexity of its pathogenesis. Disease progression is not driven solely by PrP^Sc^ accumulation, but also by disruption of proteostatic pathways, mitochondrial dysfunction, oxidative stress, synaptic degeneration, and dysregulated neuroimmune responses (Bradford et al., 2022; Kim et al., 2022). In particular, increasing evidence suggests that chronic activation of glial cells, especially microglia and astrocytes, contributes significantly to neuronal damage and disease progression (Bradford et al., 2022; Marella and Chabry, 2004). These mechanisms highlight that prion disease is not only a disorder of protein misfolding, but also a broader failure of cellular homeostasis and tissue-level regulation.

To date, multiple therapeutic strategies have been explored, including small molecules, anti-prion compounds, and symptomatic agents, yet none have shown sufficient efficacy in human clinical trials (Miranda et al., 2022). These failures reflect several major challenges, including delayed diagnosis, poor blood–brain barrier penetration, systemic toxicity, and the difficulty of targeting a self-propagating conformational pathogen (Appleby and Lyketsos, 2011; Hermann and Zerr, 2022; Dohgu et al., 2004; Shim et al., 2022). However, recent developments in prion therapeutics have begun to shift the field towards more mechanism-based strategies, including the reduction of PrP^C^ availability, immunotherapeutic targeting of prion protein, and modulation of downstream neurodegenerative processes (Shim et al., 2022). These advances suggest that a broader and more integrated therapeutic framework may be required to meaningfully alter disease course.

Among downstream therapeutic targets, neuroinflammation has emerged as a particularly important area of interest. Glial cells, including microglia and astrocytes, both exhibit a dual and context-dependent role in prion disease: in early stages they may exert protective effects through clearance of misfolded protein and maintenance of tissue homeostasis, whereas prolonged activation at later stages promotes cytokine release, oxidative stress, synaptic dysfunction, and neurotoxicity (Bradford et al., 2022; Kim et al., 2022; Marella and Chabry, 2004). This functional transition suggests that therapeutic strategies aimed at preserving protective microglial activity while limiting maladaptive inflammatory signaling may hold translational value. More broadly, interventions that combine modulation of neuroimmune responses with direct targeting of prion propagation may offer greater therapeutic benefit than single-pathway approaches.

This review examines current therapeutic strategies in prion disease, with particular emphasis on molecular targets arising from prion pathogenesis, including PrP-directed approaches, proteostatic pathways, and neuroimmune modulation. By integrating current evidence across experimental and translational studies, this review aims to evaluate the present therapeutic landscape in prion disease and identify key priorities for future intervention.

## 2 Methods

### 2.1 Review design

The aim of this scoping/narrative review was to provide a critical and interpretive overview of the molecular mechanisms underlying prion disease pathogenesis and the current therapeutic landscape, with particular emphasis on proteostatic dysfunction, neuroinflammation, glial activation, and emerging intervention strategies. This review was not designed as a formal systematic review and does not claim full PRISMA 2020 compliance (Page et al., 2020). However, selected PRISMA-inspired elements were incorporated to improve transparency of literature identification and selection as described below.

### 2.2 Search strategy

Literature searches were performed using PubMed,^1^ Web of Science,^2^ and Google Scholar^3^ to identify relevant studies on prion disease mechanisms, diagnosis, and therapy. The search covered literature published between 1995 and 2026, with earlier landmark studies included selectively where they were considered historically important or foundational to the field. The final search was conducted on 07/04/2026.

Searches combined terms related to prion biology, clinical disease, and therapeutic intervention. Core search concepts included:

“prion disease” OR “Creutzfeldt–Jakob disease” OR “transmissible spongiform encephalopathy.”

“PrPSc” OR “PrPC” OR “prion protein.”

“microglia” OR “astrocyte” OR “neuroinflammation.”

“autophagy” OR “proteostasis” OR “mitochondrial dysfunction.”

“therapy” OR “treatment” OR “clinical trial” OR “intervention.”

“PrP-lowering” OR “anti-PrP antibody” OR “gene therapy.”

Boolean operators (AND, OR) were used to refine searches. Representative search strings included:

PubMed:

(“prion disease” OR “Creutzfeldt-Jakob disease” OR “transmissible spongiform encephalopathy”) AND (“therapy” OR “treatment” OR “clinical trial” OR “intervention”)

PubMed:

(“prion disease” OR “Creutzfeldt-Jakob disease”) AND (“microglia” OR “astrocyte” OR “neuroinflammation”)

PubMed:

(“prion disease” OR “Creutzfeldt-Jakob disease”) AND (“autophagy” OR “neuroprotection”)

Equivalent searches were adapted for Web of Science and Google Scholar. Reference lists of key articles and relevant reviews were also screened manually to identify additional eligible studies.

### 2.3 Eligibility criteria

Articles were considered eligible if they addressed one or more of the following areas:

Molecular or cellular mechanisms of prion disease pathogenesis, including prion propagation, proteostatic failure, neuroinflammation, glial activation, or neuronal injury.

Diagnostic, clinical, or epidemiological aspects of human prion disease relevant to therapeutic development.

Historical or emerging therapeutic interventions in prion disease, including pharmacological, immunotherapeutic, or gene-targeting approaches.

Primary research articles were prioritized when making mechanistic or therapeutic claims. Review articles were used primarily for background context, historical framing, and identification of additional primary studies, but were not treated as equivalent to primary evidence in the core synthesis. This distinction was applied to reduce the risk of citation circularity and double counting. Studies were excluded if they were not published in English, lacked sufficient methodological detail, or were not directly relevant to prion disease or disease-relevant therapeutic mechanisms.

### 2.4 Study selection and data handling

**Figure 1:**
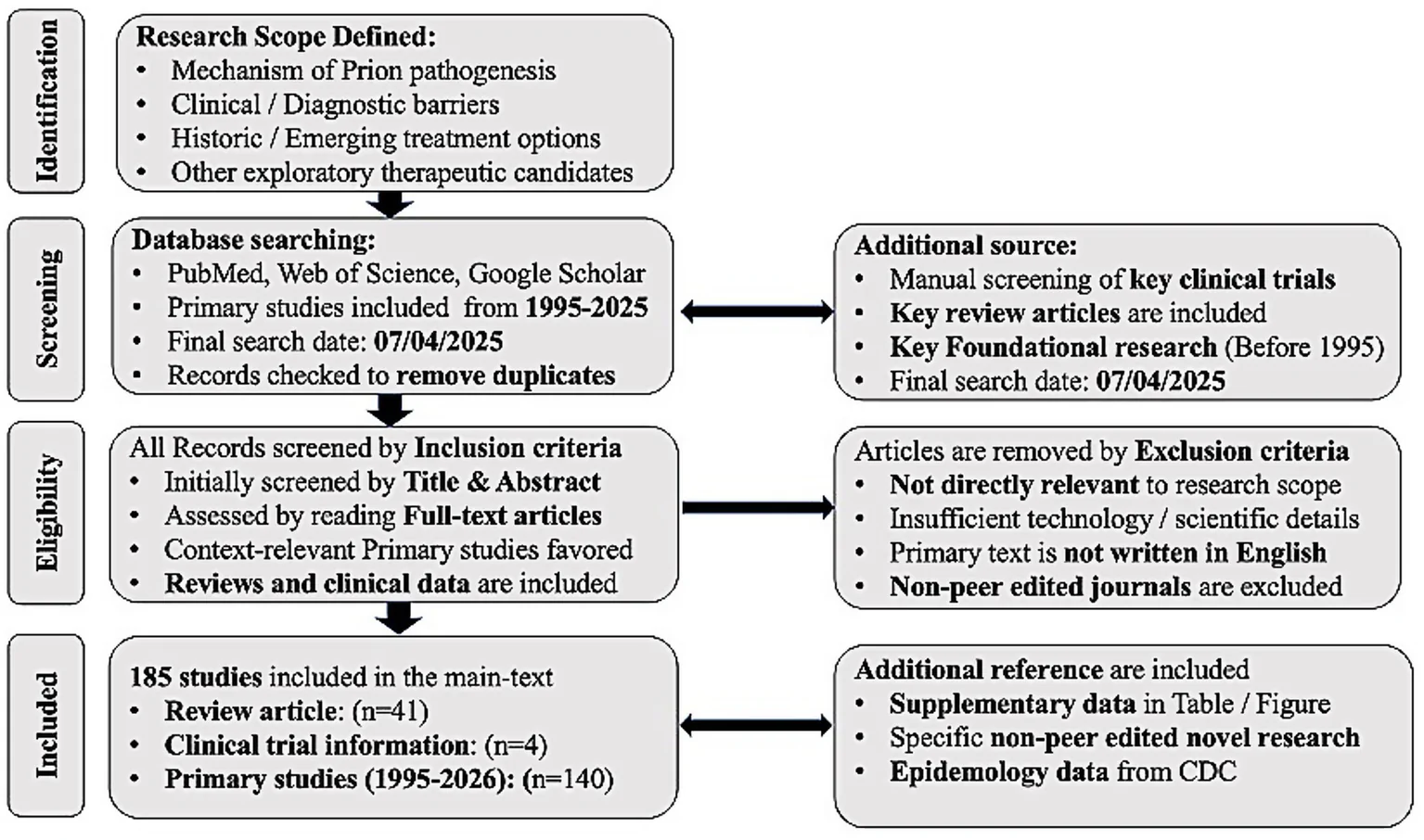
Review workflow and data extraction/screening process. Approach used to identify, screen and include studies on prion disease pathogenesis and therapeutics.

Titles and abstracts were screened for relevance. Potentially relevant records then underwent full-text review. Data extraction was performed and focused on study type, disease model or patient population, therapeutic intervention where applicable, principal mechanistic findings, and major limitations (Figure 1).

### 2.5 Approach to evidence synthesis

Evidence was synthesized narratively rather than quantitatively. The synthesis was structured around four broad areas:

1 Molecular mechanisms of prion pathogenesis;
2 Clinical and diagnostic barriers relevant to therapeutic development;
3 Historical and emerging therapeutic strategies in prion disease;
4 Downstream pharmacological modulation of disease-relevant pathways.

**Figure 2:**
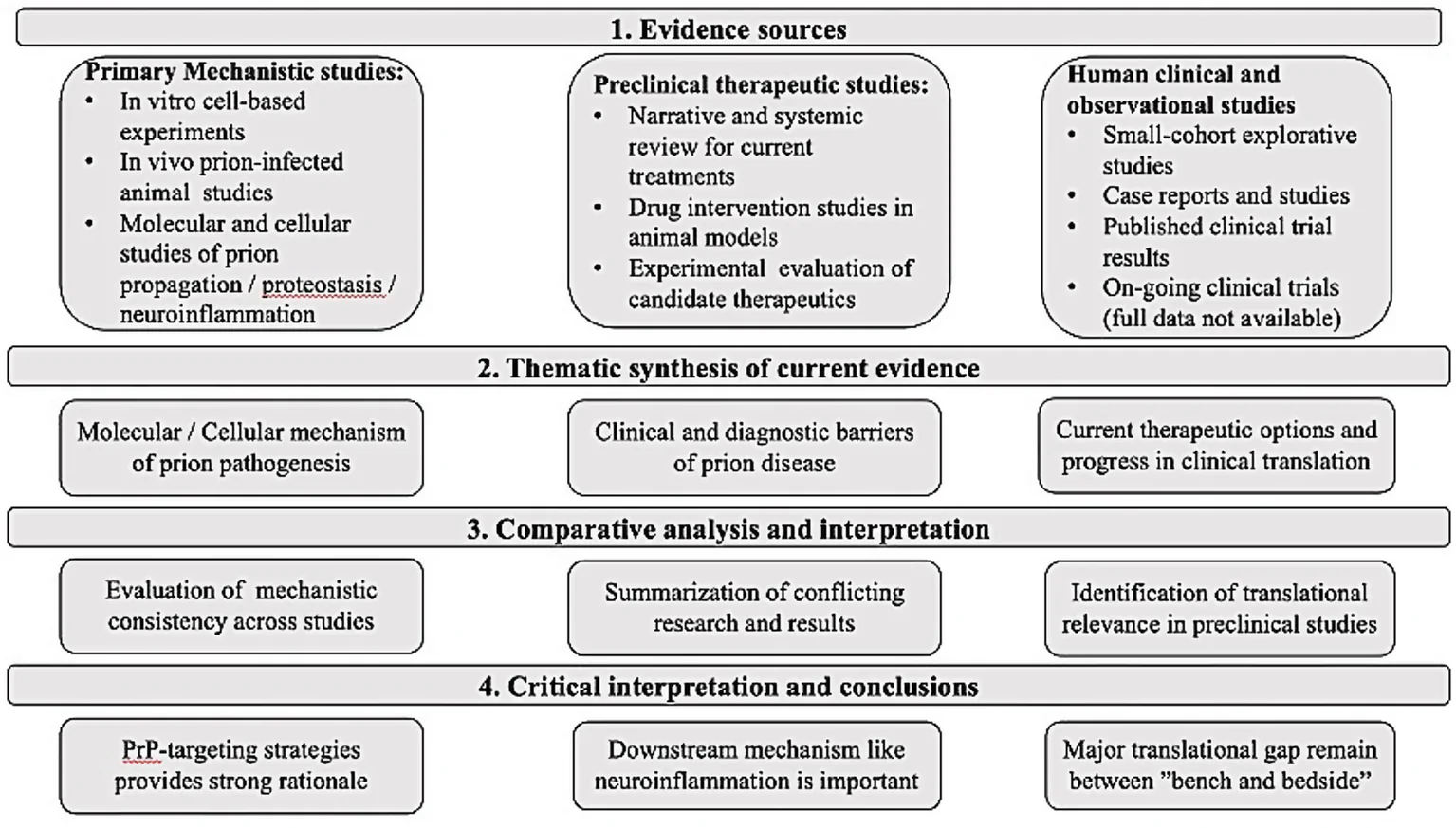
Evidence synthesis framework for the review. The review integrated mechanistic, preclinical therapeutic, and human clinical or observational evidence. These sources were synthesized into three themes: molecular and cellular mechanisms of prion pathogenesis, clinical and diagnostic barriers, and therapeutic options with translational progress. Comparative interpretation was then used to assess mechanistic consistency, conflicting findings and translational relevance.

The review aimed to identify major mechanistic themes, areas of consensus, conflicting findings, and key translational gaps across the literature (Figure 2). No formal risk-of-bias tool was applied, and no claim is made that selection bias was minimized through systematic-review safeguards. Accordingly, the conclusions of this review should be interpreted as a structured narrative synthesis rather than a systematic evaluation of the field.

## 3 Current evidence

### 3.1 Molecular mechanisms of prion pathogenesis

Prion diseases are driven by the conformational conversion of the normal cellular prion protein (PrP^C^) into the pathogenic, β-sheet-rich isoform PrP^Sc^, which acts as a template for further misfolding and propagation (Johnson, 2005). However, recent cryo-EM evidence shows that this description is an oversimplification, as infectious PrP^Sc^ is organized into precise strain-specific fibril architectures rather than being defined only by increased β-sheet content. This self-amplifying process underlies the progressive accumulation of PrP^Sc^ within the central nervous system and is associated with synaptic dysfunction, neuronal loss, and spongiform degeneration.

A central feature of prion pathogenesis is the disruption of proteostatic mechanisms that normally maintain protein homeostasis (Johnson, 2005; Chen et al., 2022). Under physiological conditions, misfolded proteins are cleared through coordinated activity of the ubiquitin–proteasome system and autophagy–lysosomal pathways. In prion disease, these systems become progressively impaired, resulting in inefficient clearance and accumulation of PrP^Sc^ aggregates (Chen et al., 2022). Autophagic dysfunction has been implicated not only in the intracellular persistence of pathogenic prion protein, but also in its propagation, and restoration of autophagic flux has been shown to reduce PrP^Sc^ burden and delay disease progression (Thellung et al., 2018). These observations identify proteostasis as a major therapeutic entry point and support the development of interventions aimed at enhancing cellular clearance pathways.

In parallel, neuroinflammatory responses play a significant role in shaping disease progression. As PrP^Sc^ progressively accumulates, both microglia, the resident immune cells of the central nervous system, and astrocytes, key regulators of neuronal inflammatory signaling, become activated and display context-dependent functional states that shape neuroinflammatory responses during prion disease. In early disease, microglia may contribute to tissue homeostasis and partial clearance of misfolded protein, whereas sustained activation promotes the release of pro-inflammatory cytokines, reactive oxygen species, and neurotoxic mediators that exacerbate synaptic dysfunction and neuronal injury (Bradford et al., 2022; Kim et al., 2022; Marella and Chabry, 2004). This transition from protective to maladaptive glial activity is further amplified through cross-talk with astrocytes, contributing to a broader failure of tissue homeostasis.

**Figure 3:**
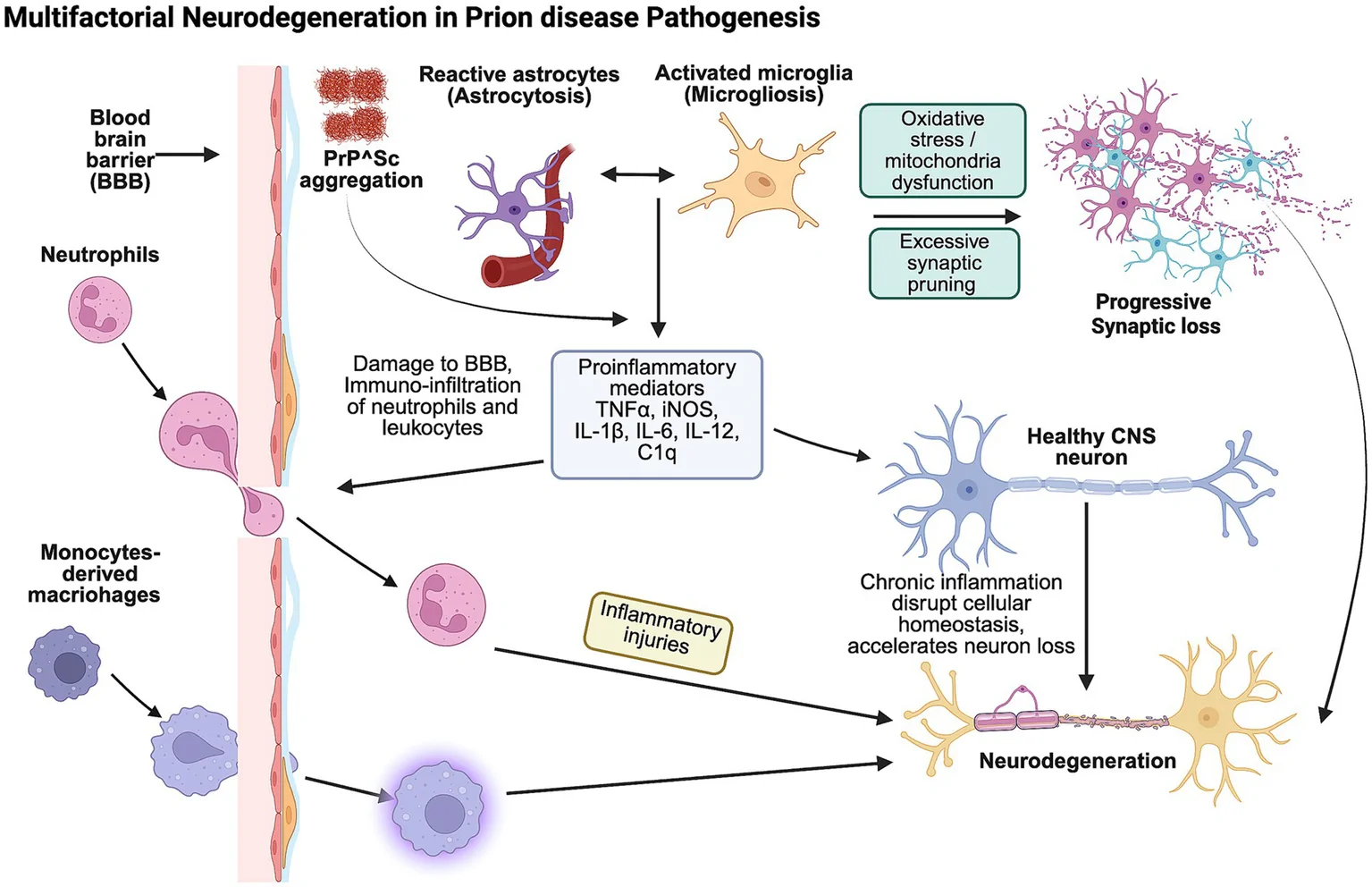
Multifactorial neurodegeneration in prion disease pathogenesis. PrP^Sc^ aggregation promotes reactive astrogliosis and microgliosis, leading to the release of proinflammatory mediators. These responses contribute to oxidative stress, mitochondrial dysfunction, excessive synaptic pruning and progressive synaptic loss. Blood–brain barrier disruption may also allow peripheral immune-cell infiltration, further amplifying inflammatory injury. Together, these interconnected processes disrupt CNS homeostasis and drive neuronal loss, illustrating why prion disease may require therapies targeting both PrP propagation and downstream tissue damage. created in BioRender. Zhu, Y. (https://BioRender.com/s9ypuiu) is licensed under CC BY 4.0.

In addition to protein aggregation and neuroinflammation, prion-induced neurodegeneration is associated with mitochondrial dysfunction, oxidative stress, and progressive disruption of synaptic integrity (Johnson, 2005; Bradford et al., 2022). Together, these processes establish a pathogenic cycle in which impaired clearance of misfolded protein enhances inflammatory activation, while chronic inflammation further disrupts cellular homeostasis and accelerates neuronal loss. Taken together, these mechanisms indicate that prion disease is not a single-pathway disorder, but rather a multifactorial neurodegenerative process requiring therapeutic strategies that address both prion propagation and downstream tissue damage (Figure 3). Therefore, approaches aimed solely at reducing PrP^Sc^ accumulation may be insufficient if secondary processes such as glial dysregulation, oxidative injury, mitochondrial impairment, and synaptic degeneration continue to drive neuronal dysfunction. Effective intervention may instead require combination or multi-target strategies that limit prion replication while also preserving tissue homeostasis and reducing neuroinflammatory and neurodegenerative damage.

### 3.2 Clinical spectrum, diagnostic challenges, and emergence of novel biomarkers in prion disease

Prion diseases affect multiple mammalian species, with animal disorders such as chronic wasting disease (CWD), scrapie, and bovine spongiform encephalopathy (BSE) highlighting the complexity of prion transmission, strain diversity, and host susceptibility (Barria et al., 2014; Brandel et al., 2000; Gallardo and Delgado, 2021; Hannaoui et al., 2021; Cassmann and Greenlee, 2020; Van Keulen et al., 2000; Smith and Bradley, 2003; Hill et al., 1997; Moore et al., 2005). In humans, Creutzfeldt–Jakob disease (CJD) represents the most common prion disorder and includes sporadic, genetic, iatrogenic, and variant forms (Gambetti et al., 2003; Iwasaki, 2017; Uttley et al., 2020; Watson et al., 2021). Despite differences in origin, these diseases share a rapidly progressive neurodegenerative course, with median survival typically less than 1 year after symptom onset (Mhawech, 2005). This rapid progression represents a major barrier for therapeutic intervention, as substantial neuronal loss and irreversible pathological changes are usually established before clinical diagnosis (Knight, 2020; Connor et al., 2019; Zerr and Poser, 2002; Heinemann et al., 2008).

Current diagnosis relies on a combination of clinical assessment, genetic testing, neuroimaging, and laboratory biomarkers. Identification of pathogenic *PRNP* variants is important for patient stratification, particularly in inherited prion diseases, whereas MRI, EEG, and cerebrospinal fluid (CSF) biomarkers provide supportive evidence of disease progression (Gambetti et al., 2003; Knight, 2020; Demaerel et al., 1999; Matsubayashi et al., 2020; Mhawech, 2005; Skillbäck et al., 2014). Most conventional CSF biomarkers, including 14–3-3 protein and total tau, reflect downstream neuronal injury rather than prion-specific pathology and therefore lack sufficient specificity for therapeutic decision-making (Mhawech, 2005; Skillbäck et al., 2014). In contrast, real-time quaking-induced conversion (RT-QuIC) has substantially improved diagnostic accuracy by detecting prion seeding activity directly, although its sensitivity remains influenced by disease subtype, sample quality, disease stage, and technical factors (Chatzikonstantinou et al., 2021; Kishida et al., 2023). Beyond diagnosis, biomarkers are increasingly important in therapeutic development by enabling patient stratification, pharmacodynamic assessment, monitoring of target engagement, and evaluation of biological treatment response (Collinge et al., 2009; Collins et al., 2002). However, changes in biomarker levels cannot be assumed to represent clinical benefit, and molecular evidence of target modulation must ultimately be linked to functional outcomes and survival. Therefore, improving early diagnosis and developing reliable biomarkers remain critical priorities for expanding the therapeutic window and enabling more effective clinical trials.

### 3.3 Repurposed small-molecule therapies in prion disease: current developments

#### 3.3.1 Therapeutic failure and the challenge of disease modification in human prion disease

Despite decades of preclinical investigation, no licensed disease-modifying therapy is currently available for CJD or other human prion diseases (Miranda et al., 2022). Early therapeutic efforts largely focused on repurposed small molecules identified through *in vitro* anti-prion activity or partial efficacy in experimental models (Appleby and Yobs, 2018). However, candidates such as quinacrine, doxycycline, pentosan polysulphate (PPS), and flupirtine illustrate the major translational barriers in prion drug development (Appleby and Yobs, 2018; Barret et al., 2003; Collinge et al., 2009; Geschwind et al., 2013). Quinacrine demonstrated reproducible inhibition of PrP^Sc^ accumulation in infected cell models but failed to improve outcomes in animal studies and clinical trials, highlighting the limited predictive value of simplified *in vitro* systems and the potential for drug-resistant prion conformers to emerge under therapeutic pressure (Barret et al., 2003; Collinge et al., 2009; Geschwind et al., 2013). Doxycycline showed anti-aggregation activity and modest efficacy in selected animal models, but inconsistent effects across prion strains and models, combined with a negative Phase II clinical trial in CJD, indicated that experimental activity did not translate into meaningful clinical benefit (Varges et al., 2017; Haïk et al., 2014; De Luigi et al., 2008; Tremblay et al., 1998; Forloni et al., 2002; Schmitz et al., 2016; Schmitz et al., 2016; Lucchetti et al., 2019; Lavigna et al., 2021; Clinical Drug Experience Knowledgebase, 2026). Potential contributors to treatment failure include late intervention after irreversible neuronal damage, inadequate CNS exposure, and the absence of reliable pharmacodynamic biomarkers to confirm target engagement (Clinical Drug Experience Knowledgebase, 2026). Similarly, PPS demonstrated anti-prion activity in cellular and animal models but was limited by strain-dependent efficacy, poor blood–brain barrier penetration requiring invasive intraventricular delivery, and insufficient clinical evidence from small observational studies (Newman et al., 2014; Bone et al., 2008; Doh-ura et al., 2004; Caughey and Raymond, 1993; Yamasaki et al., 2014; Takatsuki et al., 2022; Larramendy-Gozalo et al., 2007; Tsuboi et al., 2009). Flupirtine, which primarily targeted downstream neuronal injury rather than prion propagation itself, showed limited cognitive benefit in a small clinical study but lacked convincing preclinical validation and demonstrated no survival improvement (Otto et al., 2004; Perovic et al., 1997). Collectively, these findings demonstrate that neither suppression of prion accumulation in experimental systems nor symptomatic neuroprotection alone is sufficient to establish therapeutic efficacy.

#### 3.3.2 Efavirenz: a promising repurposed candidate targeting CYP46A1 and brain cholesterol homeostasis in prion pathogenesis

A recent example of drug repurposing in prion disease is efavirenz, an FDA-approved non-nucleoside reverse transcriptase inhibitor originally developed for the treatment of HIV infection (Ali et al., 2025). Unlike earlier repurposed compounds selected primarily on the basis of direct anti-prion effects in simplified *in vitro* systems, the therapeutic rationale for efavirenz is more mechanistically focused. Efavirenz acts as an allosteric activator of cholesterol 24-hydroxylase (CYP46A1), the key neuronal enzyme responsible for converting cholesterol into 24S-hydroxycholesterol and thereby promoting cholesterol turnover in the brain (Lerner et al., 2022). Because cholesterol homeostasis is closely linked to lipid raft integrity, membrane trafficking, and the subcellular localization of PrP^C^, dysregulation of this pathway has been proposed to contribute to prion propagation and neurodegeneration (Lerner et al., 2022; Mast et al., 2014). In this context, pharmacological activation of CYP46A1 offers a mechanistically distinct strategy aimed at modifying the lipid environment that supports prion replication rather than directly targeting PrP^Sc^ itself (Ali et al., 2025).

Preclinical studies have provided encouraging, although still limited, evidence for this approach. Ali et al. reported that efavirenz treatment reduced PrP^Sc^ accumulation in prion-infected neuronal cells and significantly prolonged survival in scrapie-infected mice, supporting the view that modulation of brain cholesterol metabolism can influence disease progression (Ali et al., 2021). The same group extended these findings to a more disease-relevant model of human prion disease, showing that low-dose oral efavirenz treatment slowed progression and extended survival in tg650 mice expressing human PrP^C^ after inoculation with sporadic CJD prions (Ali et al., 2025). Mechanistically, these effects were linked to restoration of CYP46A1 activity and altered cholesterol turnover in the brain, suggesting that impairment of this pathway may represent a previously underappreciated component of prion pathogenesis (Ali et al., 2025). Importantly, these studies imply that efavirenz does not act by lowering PrP^C^ expression directly, but rather by modifying a cellular environment permissive for prion replication and downstream neurotoxicity.

From a translational perspective, efavirenz shows promising therapeutic potential. It is already licensed for human use, crosses the blood–brain barrier, and has an established pharmacokinetic and safety profile in long-term clinical practice outside prion disease (NCT07482084). These features make it a comparatively attractive candidate for rapid clinical repurposing. Consistent with this, efavirenz has now entered formal clinical evaluation in sporadic CJD patients (NCT07482084). ClinicalTrials.gov lists an ongoing multicenter, randomized, double-blind, placebo-controlled trial designed to assess the efficacy and safety of efavirenz in patients with CJD, with survival as a key clinical outcome. This is noteworthy because it places efavirenz among the very small number of prion therapeutics to progress from experimental efficacy into structured human testing (ClinicalTrials.gov, n.d.-b) (NCT07482084).

However, the current evidence base remains preliminary, and several limitations should be acknowledged. First, the therapeutic data supporting efavirenz are still derived predominantly from preclinical models, including scrapie-based murine models and transgenic “humanized” mice expressing human PrP^C^, and it remains uncertain whether these findings will translate into meaningful clinical benefit in human CJD patients (Ali et al., 2025; Ali et al., 2021). This is particularly important because numerous compounds that were effective in scrapie-infected mice have failed when tested in human prion disease. Second, although efavirenz targets a pathway distinct from direct PrP binding or PrP lowering, the extent to which modulation of cholesterol metabolism alone can suppress established prion replication in advanced symptomatic disease remains unclear (Lerner et al., 2022). Third, efavirenz is associated with known neuropsychiatric adverse effects in other clinical contexts, and while low-dose regimens may mitigate this risk, tolerability in patients with rapidly progressive neurodegeneration has not yet been established (Abah et al., 2015; Muche et al., 2020; Ma et al., 2016). Thus, while efavirenz represents one of the more promising repurposed small-molecule strategies currently under evaluation, its therapeutic significance remains provisional pending peer-reviewed clinical outcome data.

Overall, these historical and contemporary examples reveal several recurring barriers in prion drug development (Table 1). Most candidate compounds have been introduced at advanced stages of disease, when widespread synaptic dysfunction, neuronal loss, and glial activation are already firmly established. In addition, many agents that showed promising preclinical effects were limited by poor blood–brain barrier penetration, inadequate bioavailability, or unacceptable toxicity at concentrations required for efficacy. Further, the conformational and strain-dependent nature of PrP^Sc^ propagation presents an unusually difficult therapeutic target, since the disease process is sustained by host protein conversion rather than by a conventional enzymatic pathway. Finally, even partial suppression of PrP^Sc^ accumulation may prove insufficient once downstream pathological processes, including neuroinflammation, oxidative stress, and circuit-level degeneration, have become self-reinforcing. Collectively, these limitations have shifted the field away from reliance on repurposed small molecules alone and towards more mechanism-based therapeutic strategies.

**Table 1:** Summary of representative therapeutic candidates investigated for prion disease.

Compound	Key *in vitro*/mechanistic evidence	Key preclinical studies	Key clinical research	Summarization
Quinacrine	Inhibits PrP^Sc^ formation in scrapie-infected neuroblastoma cells, but may induce drug resistant prions (Ryou et al., 2003, Ghaemmaghami et al., 2009)	Failed to prolong survival or reduce splenic PrP^Sc^, in murine prion disease model (Barret et al., 2003; Collins et al., 2002; Doh-ura et al., 2004). No effect in naturally scrapie-infected sheep (Gayrard et al., 2005)	In several phase-2 clinical trials, oral dosage at 300 mg/day, show no difference in survival or functional improvement in late-stage CJD patients (Collinge et al., 2009; Geschwind et al., 2013).	Limited anti-prion activity, but results from animal studies were largely negative. Further clinical trials found no meaningful survival benefits.
Doxycycline	Interferes with prion aggregation, caused greater than 90% reduction in protease-resistant PrP^Sc^ in *in vitro* assays (De Luigi et al., 2008; Forloni et al., 2002).	Prolonged survival in 263 K scrapie-infected hamsters, improved memory in Fatal familial insomnia (FFI) mice (De Luigi et al., 2008; Forloni et al., 2002, Lavigna et al., 2021).	In a phase-2 clinical trial, oral dosage at 100 mg/day, no improvement in survival (Haïk et al., 2014) In another clinical trial, similar oral dosage used as prevention for FFI, no formal results (Clinical Drug Experience Knowledgebase, 2026)	Anti-prion effect in cell culture and some animal models. However, no clinical trials show therapeutic effects, requiring further updates.
Pentosan polysulphate	Inhibited PrP^Sc^ in scrapie-infected neuroblastoma cells, may induce persistent infection in other prion strain infected cell lines (Caughey and Raymond, 1993; Yamasaki et al., 2014; Takatsuki et al., 2022).	Intravascular infusion extend survival by 2.4-fold in T87 mice, but show reduced survival benefit in other models, suggesting strain-dependent effects (Doh-ura et al., 2004; Larramendy-Gozalo et al., 2007).	Observational studies show that infusion modestly extend survival in some CJD patients (Bone et al., 2008; Tsuboi et al., 2009). Post-mortem study shows that it may reduce peripheral PrP accumulation (Newman et al., 2014).	Anti-prion activity, but no large-scale clinical trials, small-scale studies implicate limited extension in survival time.
**Flupirtine**	Reduced neuronal injury and apoptotic DNA fragmentation in cell cultures exposed to prion peptides (Perovic et al., 1997; Perovic et al., 1995)	No documented animal-based studies, lacking convincing *in vivo* data.	In a phase-2 clinical trial, oral dosage showed improved cognition and delayed dementia onset, but no survival benefits (Bone et al., 2008).	Showed limited anti-prion activity, primarily supported by *ex vivo* models. Its efficacy in animal remained understudied.
**Efavirenz**	Modulates cholesterol metabolism, indirectly reduced PrP^Sc^ propagation without disrupting PrP^C^ or membrane integrity (Ali et al., 2021).	Oral administration reduced PrP^Sc^ accumulation and prolonged survival in prion-infected mice (Ali et al., 2025; Ali et al., 2021).	Recently entered a phase-2 clinical trial, aim to evaluate safety and efficacy, with no data currently available or published (ClinicalTrials.gov, n.d.-b)	Showed anti-prion activity via modulation of cholesterol mechanism in brain. More animal studies are demanded, while clinical trials remain ongoing.

This table summarizes the experimental and clinical evidence for selected therapeutic candidates evaluated in prion disease, including quinacrine, doxycycline, pentosan polysulphate, flupirtine, and efavirenz. For each compound, key findings from in vitro studies, in vivo preclinical models, and human clinical research or clinical trials are compared.

### 3.4 Emerging treatment options: *PRNP-*targeted therapeutic strategies

#### 3.4.1 Therapeutic rationale for targeting PrP^C^ substrate reduction

Therapeutic development has shifted from repurposed small molecules towards mechanism-based approaches that target the central drivers of the disease. Among these, reduction of cellular prion protein (PrP^C^) expression has emerged as one of the most conceptually attractive strategies. The rationale is biologically direct. Because PrP^C^ is the obligate host substrate required for conversion into PrP^Sc^, lowering its abundance is expected to directly restrict prion replication irrespective of strain-specific conformational differences (Johnson, 2005). This substrate-lowering principle is strongly supported by early genetic evidence showing that mice lacking PrP, due to deficiency in the *Prnp* gene which encodes PrP^C^, are resistant to scrapie infection, demonstrating that host PrP^C^ expression is indispensable for disease susceptibility (Manson et al., 1994; Büeler et al., 1993). Collectively, these findings provided the mechanistic basis for therapeutic strategies aimed at suppressing PrP protein or gene expression as a means of preventing or slowing disease progression.

#### 3.4.2 Antisense oligonucleotides: progress in pharmacological suppression of *PRNP* expression and recent clinical application of ION717

One of the most important translational advances in prion therapeutics has been the development of antisense oligonucleotides (ASOs) to reduce PrP gene expression *in vivo* (Nazor Friberg et al., 2012). ASOs are short, synthetic single-stranded nucleic acid molecules designed to bind complementary RNA transcripts through mechanisms such as transcript degradation, altered splicing, or translation inhibition (Nazor Friberg et al., 2012). Nazor Friberg et al. (2012) demonstrated that intracerebral delivery of ASOs could lower *Prnp* mRNA expression in the mouse brain, providing initial proof-of-concept evidence that pharmacological PrP suppression was achievable within the CNS (Hill et al., 1997). Raymond et al. (2019) subsequently reported prolonged survival in prion-infected mice following ASO treatment in both prophylactic and delayed-dosing paradigms (Raymond et al., 2019). The observation that benefit persisted when treatment was initiated after infection provided important evidence that PrP lowering could modify disease progression after prion replication had begun (Raymond et al., 2019). Minikel et al. (2020) further showed that *Prnp*-targeting ASOs exerted disease-modifying effects across multiple prion strains and disease stages, reinforcing the robustness of substrate reduction as a therapeutic principle. Nevertheless, these preclinical studies were conducted predominantly in experimentally infected rodent models, in which disease onset, inoculation timing, and treatment schedules can be tightly controlled. Such conditions differ substantially from human prion disease, where diagnosis usually occurs after neurological symptoms emerge and the duration of presymptomatic prion replication remains unknown. The magnitude of benefit observed in rodents may therefore overestimate the effect achievable in symptomatic patients.

Large-animal studies have strengthened the feasibility of CNS PrP-lowering but have not yet demonstrated therapeutic efficacy in a large-animal prion disease model. Mortberg et al. (2023) used single-cell transcriptomic approaches to characterize ASO distribution in mouse and non-human primate brain and spinal cord, showing broad *PRNP* knockdown across multiple cell types. Centrally administered ASOs were associated with an approximately 40% reduction in cerebrospinal fluid PrP, supporting CSF PrP as a clinically relevant pharmodynamic biomarker of target engagement (ClinicalTrials.gov, n.d.-c). However, these data primarily evaluated ASO distribution and molecular response rather than effects on prion replication, neurological decline or survival. Rather, these data indicate that widespread CNS PrP suppression is achievable beyond rodent systems, thereby strengthening the case for clinical translation while also highlighting the need for more extensive preclinical validation.

ION717 is the first *PRNP*-targeting ASO to enter formal clinical evaluation in patients with prion disease (NCT06153966). It is designed to bind *PRNP* mRNA and reduce the synthesis of cellular prion protein, thereby limiting the substrate required for continued PrP^Sc^ amplification (ClinicalTrials.gov, n.d.-c). Mechanistically, this strategy is especially attractive because it does not aim to neutralize already accumulated PrP^Sc^ directly, but rather to reduce the availability of host PrP^C^ required for continued prion amplification. In this way, ION717 may offer a more biologically upstream intervention than conventional small-molecule therapies, which have largely targeted downstream stress responses or attempted to interfere with pathogenic aggregates after substantial neuropathology was already established.

The ongoing PrProfile study is a first-in-human Phase 1/2a clinical trial designed to evaluate the safety, tolerability, pharmacokinetics, and pharmacodynamics of intrathecal delivery of ION717 (ClinicalTrials.gov, n.d.-c; European Union Clinical Trials Register, n.d). Its randomized and blinded treatment period, followed by longer-term follow-up, is appropriate for an early-stage programme focused principally on feasibility, safety, and biological target engagement rather than definitive efficacy. Progression into clinical testing represents an important translational milestone, as it moves from rodent and non-human-primate studies into human investigation (ClinicalTrials.gov, n.d.-c). However, the trial design and current development stage mean that conclusions regarding disease modification, preservation of neurological function, or survival benefit cannot yet be drawn. Enrolment for the PrProfile study was reported to have been completed in late 2024 and the study was subsequently expanded to include an additional dosing regimen following preliminary analysis of early trial data (European Union Clinical Trials). Although these developments suggest continued sponsor confidence, it is important to emphasize that such updates do not constitute peer-reviewed efficacy evidence. At the time of writing (June 2026), the available information established only that ION717 remained under active clinical investigation and that longer-term exposure data were being collected through extension phases and modified dosing schedules (European Union Clinical Trials). No peer-reviewed clinical results had demonstrated that ION717 slows neurological decline or prolongs survival in patients with symptomatic prion disease.

Although ION717 represents an important translational advance from preclinical studies, several limitations should be acknowledged. The lack of early diagnostic tools may limit the efficacy of ION717 by delaying treatment until substantial neurodegeneration has already occurred (Wang et al., 2023). Moreover, repetitive treatment via intrathecal delivery remains invasive and may induce severe adverse effects, carrying long-term infection risk (Schreiner et al., 2025; Dario et al., 2005). Further, preclinical evaluation of PrP-lowering ASOs relies on reduced level of CSF PrP, which act as a potential pharmodynamic biomarker, rather than direct validation of outcome (Vallabh et al., 2020; Senesi et al., 2023). Conclusively. the therapeutic significance of ION717 remains provisional until peer-reviewed clinical outcome data become available.

#### 3.4.3 siRNA-mediated *PRNP* silencing: a parallel RNA-based approach showing promising therapeutic effect in early development

In addition to ASO-based PrP suppression, small interfering RNA (siRNA)-mediated targeting of *PRNP* has emerged as a parallel RNA-based strategy in early translational development (Zabel et al., 2021; Lehmann et al., 2014; White et al., 2008). Like ASOs, siRNA-based approaches aim to reduce the synthesis of cellular prion protein by targeting the *PRNP* transcript. However, they differ mechanistically in their reliance on RNA interference pathways rather than antisense-mediated transcript degradation (Dana et al., 2017; ClinicalTrial.gov, n.d.-a).

The mechanistic basis for this approach was established in early cell-based studies. Synthetic siRNA duplexes directed against Prnp induced sequence-specific gene silencing in scrapie-infected neuroblastoma cells, resulting in depletion of cellular PrP and a rapid reduction in protease-resistant pathological PrP (Daude et al., 2003; Gentile et al., 2026). Subsequent studies confirmed that Prnp-targeting siRNAs reduced PrP^C^ expression in both uninfected and prion-infected neuroblastoma cells and suppressed or eliminated detectable PrP^Sc^ in infected cultures (Kim et al., 2009). Human-sequence siRNAs were also shown to reduce endogenous PrP expression in SH-SY5Y cells and recombinant wild-type or familial CJD-associated PrP variants in HEK293T cells, supporting the applicability of the platform to human *PRNP* targets (Wang et al., 2011). However, these experiments were conducted in simplified cellular systems and primarily measured biochemical PrP reduction. They therefore establish target engagement and mechanistic plausibility but do not demonstrate that comparable knockdown can be achieved safely and uniformly across the human CNS or that reductions in detectable PrP^Sc^ correspond to durable suppression of prion infectivity.

*In vivo* studies have provided stronger evidence that siRNA-mediated PrP lowering can influence disease progression. Early work using lentiviral delivery of anti-PrP siRNA in scrapie-infected mice reduced PrP expression, rescued aspects of early neuronal dysfunction, and prolonged survival (Lehmann et al., 2014; White et al., 2008). These findings provided proof of principle that RNA interference could modify disease *in vivo*, but the use of a viral vector raises important translational concerns, including immunogenicity, irreversibility, control of tissue distribution, and long-term safety. Later non-viral approaches, including systemically administered PrP-siRNA formulations and liposome–siRNA–peptide complexes, demonstrated that PrP expression could be reduced in the mouse brain after peripheral administration (Lehmann et al., 2014; Gentile et al., 2026; Bender et al., 2019). These studies improved the practical relevance of the platform, although the magnitude and regional distribution of CNS knockdown remained variable, and evidence for consistent survival benefit was more limited than for ASO-based approaches.

More recent work with chemically optimized divalent siRNAs targeting *Prnp*/*PRNP* suggests that more durable brain PrP lowering may be achievable, with associated survival extension in prion-infected mice (Gentile et al., 2026). This represents an important advance because durability of knockdown is a major determinant of clinical feasibility for RNA-based therapies. Nevertheless, these findings remain recent, and their robustness will depend on independent replication, evaluation across different prion strains and treatment stages, and confirmation that prolonged PrP suppression does not produce unanticipated neurological or systemic toxicity. The available evidence therefore supports biological activity and therapeutic potential, but the preclinical literature remains less mature than that of PrP-lowering ASOs (Gentile et al., 2026).

Clinical translation has begun with the registration of a first-in-human, open-label, single-ascending-dose study evaluating intrathecal administration of a divalent *PRNP*-targeting siRNA in adults with symptomatic prion disease (ClinicalTrial.gov, n.d.-a). The study includes dose levels of 50, 100, and 200 mg and is designed primarily to assess safety, tolerability, pharmacokinetics, and changes in CSF PrP as a pharmacodynamic marker. This represents an important step beyond experimental models, but the open-label, early-phase design is not intended to establish clinical efficacy. No peer-reviewed data have yet shown that *PRNP*-targeting siRNA slows neurological decline, reduces prion seeding activity, or prolongs survival in patients.

Several translational uncertainties therefore remain. Efficient and sufficiently broad CNS delivery is still a major challenge, particularly because reductions in CSF PrP may not fully reflect knockdown in all disease-relevant brain regions. The durability of suppression, need for repeat dosing, and long-term safety of sustained PrP lowering also remain incompletely defined. As with ASOs, the timing of intervention is likely to be critical, because treatment initiated after extensive neuronal loss may have limited capacity to reverse established pathology. In addition, CSF PrP functions primarily as a marker of target engagement and cannot by itself establish disease modification or clinical benefit.

Overall, *PRNP*-targeting siRNA is a mechanistically compelling but still early-stage therapeutic strategy. Cell-based studies provide strong evidence that RNA interference can reduce PrP^C^ and suppress detectable PrP^Sc^, while animal studies indicate that PrP knockdown can improve neurological outcomes and prolong survival under selected experimental conditions. However, compared with ASOs, the siRNA platform has a less developed survival evidence base, greater uncertainty regarding delivery, and no published human efficacy data. Its translational significance will therefore depend on whether durable and sufficiently widespread CNS PrP suppression can be achieved safely and whether this molecular effect translates into clinically meaningful benefit in symptomatic patients.

#### 3.4.4 *PRNP* genome editing: toward durable or one-time PrP-lowering intervention

While *PRNP*-targeting ASOs and siRNAs have advanced into early clinical development, their dependence on repeated CNS administration has encouraged investigation of more durable approaches to PrP suppression. Direct genomic modification of *PRNP* is one such strategy, but it remains at a substantially earlier stage of development than RNA-based therapies (An et al., 2025; Medd and Cao, 2024; Allais-Bonnet et al., 2025). Initial studies established the technical feasibility of disrupting *PRNP* in cell-based systems. CRISPR–Cas9-mediated editing was first used to eliminate PrP expression in murine cell lines, providing proof of principle that the *PRNP* locus could be genetically modified (Mehrabian et al., 2014). Subsequent dual-guide CRISPR–Cas9 editing of exon 3 generated *PRNP*-knockout murine cell lines that were used to investigate cellular susceptibility to prion propagation (Walia et al., 2019). These studies demonstrated durable loss of PrP expression, but they were primarily experimental models rather than therapeutic interventions and did not address delivery, safety, or efficacy in an intact nervous system.

More recent work has shifted from complete gene disruption toward base-editing strategies designed to introduce premature stop codons into *PRNP*. An et al. (2025) used adeno-associated virus-delivered cytosine and adenine base editors to modify the *PRNP* locus *in vivo* (Medd and Cao, 2024). Following cell-based validation, the approach was evaluated in humanized mice inoculated with human prion disease isolates. Treatment produced a durable reduction in brain PrP expression and prolonged survival, providing preclinical evidence that permanent substrate reduction can modify disease progression *in vivo* (An et al., 2025). The use of a humanized model and human prion isolates strengthens the biological relevance of these findings compared with conventional murine scrapie systems. Nevertheless, the evidence remains restricted to a single preclinical platform, and no large-animal study or independent clinical investigation has yet demonstrated the safety, reproducibility, or therapeutic efficacy of *PRNP* editing.

The principal potential advantage of genome editing is durability. In contrast to ASOs or siRNAs, which may require repeated administration to maintain suppression, successful editing could theoretically produce sustained PrP lowering after a single treatment. However, describing this approach as a clinically realistic “one-time therapy” would currently be premature. Its translational feasibility depends on achieving sufficiently broad and uniform editing across disease-relevant regions of the CNS, while limiting off-target genomic changes, vector-related toxicity, immunogenicity, and unintended long-term consequences of irreversible *PRNP* modification (Kantor et al., 2025; Pandya and Kumar, 2026). The degree of editing required for therapeutic benefit is also unknown, and incomplete regional coverage could leave sufficient PrP expression to support continued prion propagation.

Treatment timing presents an additional difficulty. The survival benefit observed in controlled mouse experiments does not establish that genome editing would remain effective when administered after clinical diagnosis in rapidly progressive human prion disease. Vector delivery, editor expression, and genomic modification require time, whereas symptomatic CJD may progress over weeks or months. Genome editing may therefore be less suitable for late-stage rescue than for intervention before extensive neuronal loss, although this possibility has not yet been tested clinically. Inherited prion diseases represent a biologically relevant context in which durable *PRNP* suppression might eventually be considered. Genetic CJD, fatal familial insomnia (FFI), and Gerstmann–Sträussler–Scheinker syndrome (GSS) are associated with pathogenic *PRNP* variants, including E200K, V210I, V180I, M232R, D178N coupled with methionine at codon 129, and P102L (Appleby et al., 2022; Kim et al., 2018; Parchi et al., 1998). In principle, genome-editing approaches could reduce total PrP expression or selectively target a pathogenic allele in presymptomatic carriers. However, current evidence does not demonstrate mutation correction, allele-selective silencing, prevention of disease onset, or clinical benefit in individuals with inherited *PRNP* mutations. These applications should therefore be presented as possible future research directions rather than established therapeutic opportunities.

Overall, *PRNP* genome editing provides important proof of concept for durable PrP lowering, but it remains an entirely preclinical strategy. The strongest available evidence is the demonstration that AAV-delivered base editing can reduce brain PrP and extend survival in a humanized mouse model. Before clinical translation can be considered, these findings require independent replication, validation in additional prion strains and disease stages, assessment in larger animal models, and extensive evaluation of CNS distribution, off-target editing, immunogenicity, and long-term neurological safety.

### 3.5 Anti-PrP immunotherapy: preclinical and translational evidence

In parallel with substrate-lowering approaches, anti-prion immunotherapy has emerged as a promising but technically challenging therapeutic strategy (Taschuk et al., 2015; Jones et al., 2010; Peretz et al., 2001; Féraudet et al., 2005; White et al., 2003). As shown by cell-based models, Anti-PrP^C^ monoclonal antibodies may bind cellular prion protein, interfere with its conversion to PrP^Sc^, or facilitate clearance of disease-associated PrP species (Peretz et al., 2001; Enari et al., 2001; Bender et al., 2019; Perrier et al., 2004). Animal studies provided further support for passive immunotherapy. White et al. (2003) showed that anti-PrP monoclonal antibodies delayed disease onset and prolonged incubation time in prion-exposed mice (White et al., 2003). Subsequent studies extended this rationale to additional antibody platforms, including ICSM18, ICSM35, and other monoclonal antibodies directed against distinct epitope (Adhikari et al., 2021; Reilly et al., 2022; Klöhn et al., 2012; Ma et al., 2016; Makarava et al., 2019; Manson et al., 1994). Ohsawa et al. (2013) further demonstrated that peripheral administration of an anti-PrP monoclonal antibody prolonged survival in prion-infected mice, indicating that at least some degree of therapeutic efficacy might be achieved without direct intracerebral delivery. Nevertheless, therapeutic benefit varied substantially according to antibody identity, epitope specificity, administration route, dose, and timing relative to infection. Much of the strongest preclinical efficacy was observed when treatment was initiated before or soon after prion exposure, whereas relevance to treatment of established symptomatic disease remains less certain. Contradictory findings from several preclinical studies demonstrate that anti-PrP antibodies exhibit high heterogenicity and cannot be regarded as a uniformly protective therapeutic class. Antibodies within the POM series recognize non-overlapping regions of the PrP^C^ and differ markedly in their biological effects (Polymenidou et al., 2008; Gilch et al., 2003). Some antibodies suppress prion propagation, whereas others can induce acute neuronal injury. Antibodies targeting certain globular-domain epitopes, including POM1, induce acute neurotoxicity, and may activate neurotoxic pathways that converge with those triggered by prion infection itself (Reimann et al., 2016; Herrmann et al., 2015). ICSM18 showed a more favorable toxicity profile. Therapeutic development therefore requires careful selection of antibody epitope, affinity, valency, and mode of target engagement rather than assuming that all PrP-binding antibodies will be protective.

PRN100, a humanized monoclonal antibody derived from ICSM18, is the most clinically advanced anti-PrP antibody reported to date (Mead et al., 2022). In the first-in-human treatment programme described by Mead et al. (2022), PRN100 was administered intravenously to six patients with CJD under a Special License framework. The study demonstrated that repeated systemic administration was feasible, that the antibody reached pharmacologically relevant concentrations in cerebrospinal fluid, and that treatment was broadly tolerated in this small cohort. These findings addressed important questions regarding delivery and short-term safety. However, all treated patients continued to deteriorate neurologically and died from their disease. Because the programme involved only six patients, lacked a randomized control group, and treated individuals with rapidly progressive disease, it could not determine whether PRN100 produced any modest effect on functional decline or survival. The study should therefore be interpreted as evidence of feasibility and CNS exposure rather than evidence of clinical efficacy.

The negative clinical course of the treated patients does not necessarily invalidate the underlying therapeutic principle, but it highlights several translational barriers. First, antibody penetration into the CNS remains limited relative to circulating concentrations, and CSF exposure may not accurately reflect uniform target engagement throughout affected brain regions (Mead et al., 2022). Second, treatment initiated after extensive synaptic dysfunction and neuronal loss may be unable to reverse established neurodegeneration, even if further PrP conversion is partly suppressed. Third, the possibility of target-mediated neurotoxicity narrows the therapeutic window and requires more extensive safety assessment than would be necessary for antibodies directed against an inert extracellular target. Finally, no validated pharmacodynamic marker has yet shown that antibody exposure in patients results in reduced prion seeding activity or meaningful inhibition of PrP conversion (Mead et al., 2022).

A possible role for anti-PrP antibodies in presymptomatic or post-exposure settings has been proposed because several animal studies achieved their greatest benefit when treatment was initiated early (White et al., 2003; Adhikari et al., 2021; Reilly et al., 2022; Klöhn et al., 2012; Ohsawa et al., 2013). In principle, passive immunotherapy administered shortly after accidental or iatrogenic exposure might reduce peripheral prion amplification or delay neuroinvasion (Klyubin et al., 2014; Masone et al., 2025). However, this application remains untested in humans, and the available evidence does not establish that PRN100 can neutralize prions after exposure, prevent CNS invasion, or alter long-term disease risk. Practical limitations would also include identifying a credible exposure event, defining an effective treatment window, achieving adequate distribution to peripheral lymphoreticular tissues and the nervous system, and balancing uncertain benefit against repeated antibody administration. Post-exposure prophylaxis should therefore be presented as a preclinical research hypothesis rather than a clinically realistic current indication.

Overall, anti-PrP monoclonal antibodies have strong mechanistic support and reproducible evidence of anti-prion activity in cellular and selected animal models. However, their translational potential is constrained by variable efficacy across antibodies, dependence on early treatment, limited CNS penetration, and epitope-specific neurotoxicity. PRN100 represents an important translational milestone because it demonstrated that systemic anti-PrP antibody administration and measurable CNS exposure are feasible in patients. Nevertheless, no survival or functional benefit has yet been established, and future development will require antibodies with carefully validated epitope specificity, favorable neurotoxicity profiles, improved CNS delivery, and biomarkers capable of distinguishing target engagement from genuine disease modification.

Taken together, *PRNP*- and PrP-directed therapies intervene at several levels of the pathogenic process, including suppression of *PRNP* transcription, depletion of PrP^C^ substrate, durable genomic disruption, inhibition of PrP^Sc^ conversion, and antibody-mediated neutralization of PrP species (Table 2). Their evidence bases and translational readiness differ substantially. ASOs currently have the most advanced clinical development pathway, siRNA remains in early human evaluation, genome editing is restricted to preclinical proof-of-concept studies, and anti-PrP antibodies have demonstrated clinical feasibility without established efficacy.

**Table 2:** Summary of *PRNP*- and PrP-targeting therapeutic strategies in prion disease.

Strategies	Key *in vitro*/mechanistic evidence	Key *in vivo* preclinical studies/animal models	Key clinical research/clinical trials	Summarization
***PRNP*-targeting antisense oligonucleotides (ASOs)**	ASOs are designed to bind *PRNP* mRNA and reduce PrP production through RNase H1-mediated RNA degradation (Nazor Friberg et al., 2012).	Prophylactic dosing extended survival in prion-infected mice, while treatment near clinical onset also prolonged survival (Raymond et al., 2019; Minikel et al., 2020).	ION717/PrProfile trial evaluates safety and pharmacokinetics of ASOs, further data requires update (ClinicalTrials.gov, n.d.-c).	ASOs show convincing preclinical outcomes in both *in vitro* and *in vivo* models. Clinical efficacy in humans remains unproven.
***PRNP*-targeting siRNA**	Human-sequence siRNAs target *PRNP* to induce specific gene silencing, leading to depletion of cellular PrP and rapid reduction of protease-resistant pathological PrP (Daude et al., 2003; Kim et al., 2009; Wang et al., 2011).	Early *in vivo* siRNA studies used lentiviral delivery. Later non-viral delivery, such as systemic formulations and liposome peptide, are developed (Lehmann et al., 2014; White et al., 2008; Bender et al., 2019) Recently, divalent siRNA achieved durable brain PrP lowering and extended survival in mice (Gentile et al., 2026).	Intrathecal PrP-siRNA is being evaluated in adults with symptomatic prion disease. Follow-ups used to assess safety and pharmacokinetics (Gentile et al., 2026). CSF PrP concentration as a pharmacodynamic marker. However, no peer-reviewed data have yet been reported.	*PRNP*-targeting siRNA is a mechanistically strong but still early-stage therapeutic strategy. *In vitro* results provide strong mechanistic rationale. Translational efficacy remains limited and requires further research.
*PRNP* genomic editing/base editing	CRISPR–Cas9-mediated editing of *Prnp* was first validated in murine cell lines. Later, dual-guide editing was developed to achieve durable PrP lowering cell line (An et al., 2025; Mehrabian et al., 2014; Walia et al., 2019).	*PRNP* base editing in humanized mice model reduced PrP levels in brain and extended survival (An et al., 2025)	No clinical trial has yet evaluated *PRNP* genomic editing or base editing in human prion disease.	*PRNP* genomic editing remains a highly promising but entirely preclinical strategy, showing strong mechanistic rationale but lacking clinical evidences.
Anti-PrP monoclonal antibody therapy	Anti-PrP antibody inhibited PrP^Sc^ formation in scrapie-infected mouse neuroblastoma cells, clearing pre-existing PrP^Sc^ (Peretz et al., 2001; Perrier et al., 2004). Antibody efficacy differs substantially in epitope specificity.(Polymenidou et al., 2008)	Passive immunization delayed disease onset in prion-exposed mice, (White et al., 2003; Ohsawa et al., 2013). Some antibodies also showed epitope-dependent neurotoxicity, with POM1 inducing neuronal damage (Reimann et al., 2016).	PRN100, a humanised anti-PrP antibody was administered to six CJD patients. PRN100 reached pharmacologically relevant CSF concentrations, but all treated patients continued to deteriorate and died, clinical efficacy was not established (Mead et al., 2022).	Anti-PrP monoclonal antibody therapy has strong mechanistic and preclinical support. Clinical evidence remains very limited, no survival or functional benefit has yet been proven.

This table summarizes major mechanism-based therapeutic approaches that aim to reduce *PRNP* mRNA expression or directly target PrP protein availability, including *PRNP*-targeting siRNA/RNAi, *PRNP* genomic editing, and anti-PrP monoclonal antibody therapy. For each strategy, key in vitro evidence, *in vivo* preclinical findings, clinical or translational status, and overall interpretation are compared.

### 3.6 Glial cells as novel cellular therapeutic targets in prion disease

#### 3.6.1 Therapeutic modulation of microglia: opportunities and translational limitations

In addition to therapies that directly suppress PrP expression or interfere with prion propagation, glial cells represent downstream cellular targets for therapeutic intervention. Microglia and astrocytes participate in prion clearance, inflammatory signaling, synaptic maintenance, metabolic support, and neuronal injury (Bilimoria and Stevens, 2015). However, their responses are heterogeneous and may be protective or detrimental depending on disease stage and activation state. Glial-directed treatment should therefore aim to modulate specific pathological pathways while preserving beneficial homeostatic and clearance functions, rather than broadly suppressing glial activity.

**Figure 4:**
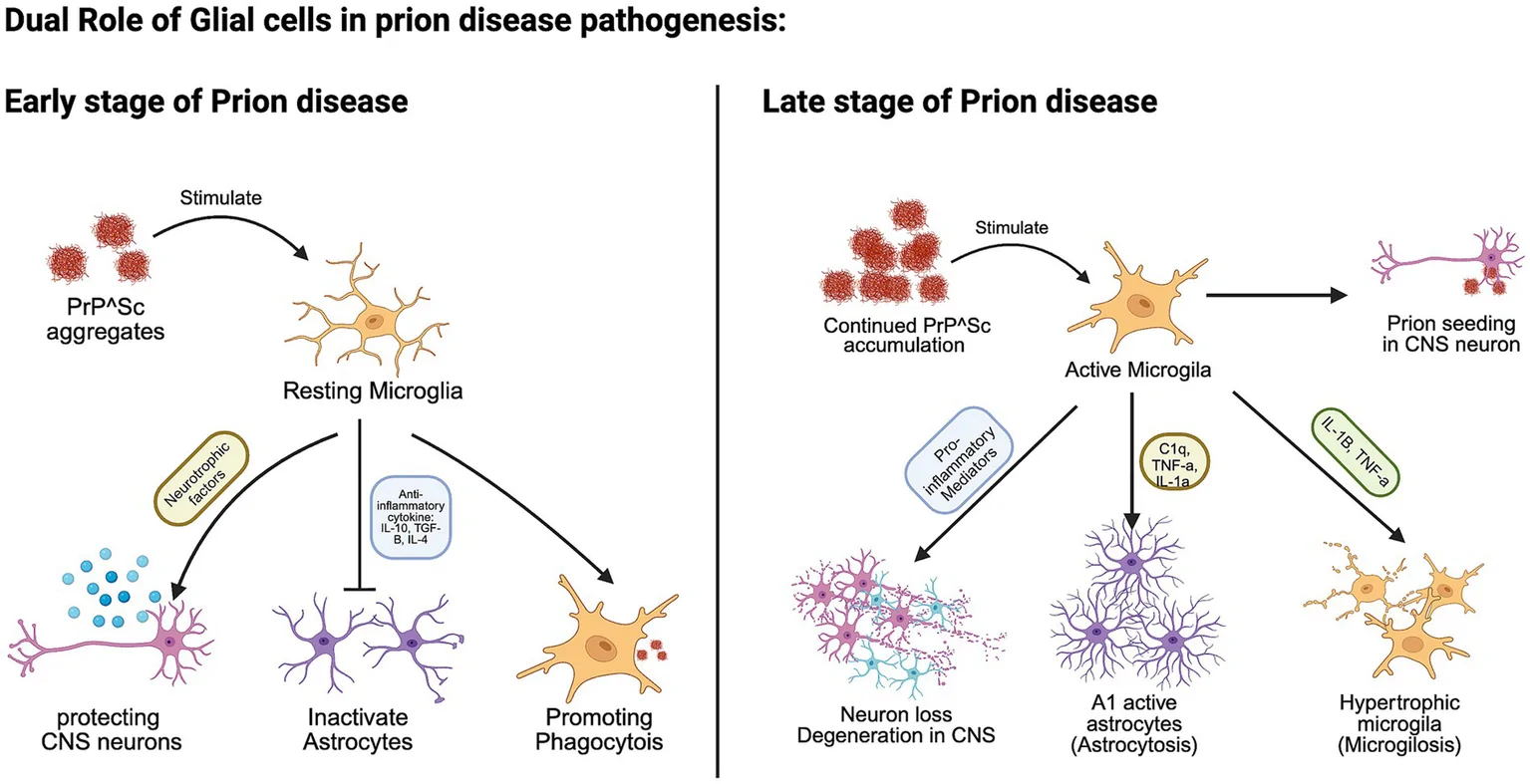
Dual role of glial cells in prion disease pathogenesis. The figure illustrates the contrasting functions of glial cells during prion infection and neurodegeneration. The left panel highlights protective glial functions, while the right panel illustrates maladaptive glial activation and neurotoxic signalling during chronic disease progression, emphasising the stage-dependent role of neuroinflammation in prion disease. created in BioRender. Zhu, Y. (https://BioRender.com/o0bnh5k) is licensed under CC BY 4.0.

Microglia, the resident parenchymal macrophages of the CNS, play essential roles in immune surveillance, synaptic remodeling, and maintenance of neuronal homeostasis (Hong et al., 2016; Bilimoria and Stevens, 2015; Prinz et al., 2019). Under physiological conditions, microglia clear apoptotic debris, prune synapses, and secrete trophic factors that support neuronal development and tissue integrity (Prinz et al., 2019). In prion disease, however, microglial function becomes profoundly altered, reflecting a context-dependent dual role in both neuroprotection and neurotoxicity (Carroll and Chesebro, 2019; Brown, 2001). During the early stages of prion infection, microglial activation is generally considered protective (Brown, 2001). In response to progressive PrP^Sc^ accumulation, microglia upregulate phagocytic and lysosomal pathways, promoting partial clearance of prion aggregates from the parenchyma (Brown, 2001; de Melo et al., 2021). Microglia also contribute to regulation of the local inflammatory response through secretion of anti-inflammatory mediators, including IL-4, IL-10, and TGF-β, which may dampen excessive neuroinflammation (Marella and Chabry, 2004; de Melo et al., 2021; Obst et al., 2017; Chen and Dong, 2025; Stoeck et al., 2005). Collectively, these findings identify microglia as central components of the early neuroprotective response to prion infection. As disease progresses, however, sustained PrP^Sc^ accumulation appears to drive microglia towards maladaptive activation states (Figure 4). Persistent microglial activation increases the production of reactive oxygen species and proteolytic enzymes, thereby amplifying local tissue injury (Gómez-Nicola et al., 2013; Peggion et al., 2020; Hay et al., 2025). Importantly, activated microglia also release C1q, TNF-*α*, and IL-1α, which promote the induction of neurotoxic reactive astrocyte states and sustain neuroinflammation (Asano et al., 2020; Liddelow et al., 2017). In this later disease context, microglia that initially restricted prion-associated damage may lose their protective capacity and instead promote pathogenesis.

Whether microglia directly facilitate prion propagation during late-stage disease remains uncertain. Microglia are not generally considered major reservoirs of PrP^Sc^ (Carroll and Chesebro, 2019; Montrasio et al., 2000; Li et al., 2021). Instead, under physiological conditions, they appear to degrade more prions than they propagate (Bradford et al., 2022). However, neuronal injury triggered by microglial cytokines and oxidative stress can promote release of intracellular PrP^Sc^ into the extracellular space (Lawson, 2024). Microglia may therefore contribute indirectly to late-stage prion accumulation by creating conditions that accelerate the secondary release of infectious material. Post-mortem neuropathological studies consistently demonstrate extensive microgliosis, often accompanied by reactive astrocytosis, supporting a close association between glial activation and disease progression. Microgliosis is characterized by microglial proliferation and hypertrophy (Monzón et al., 2018; Franceschini et al., 2018; Krbot et al., 2018; Ballweg et al., 2023; Crozet et al., 2008; Sigurdson et al., 2019). Elevated concentrations of inflammatory cytokines in the cerebrospinal fluid of symptomatic sCJD patients further indicate that maladaptive glial activation persists during the disease course (GuoYan et al., 2025; Stoeck et al., 2006).

Taken together, these studies highlight the dual and stage-dependent role of microglia in prion pathogenesis. In early disease, microglia exert neuroprotective effects; in later stages, they adopt maladaptive phenotypes that accelerate neuronal loss (Prinz et al., 2019; Brown, 2001; de Melo et al., 2021). This complexity limits the therapeutic value of indiscriminate microglial depletion or broad suppression. A clinically relevant strategy would instead require selective modulation of harmful microglial pathways while preserving phagocytic, trophic and homeostatic functions. However, the specific microglial states that should be targeted, the optimal timing of intervention, and the extent to which such modulation can alter prion replication remain incompletely defined.

More recently, microglial replacement and engineering approaches have been explored in other neurodegenerative disease models, particularly Alzheimer’s disease, where modifying CNS immune cell populations has shown potential to alter pathology (Yoo et al., 2023; Jiang and Jin, 2023; Mishra et al., 2023; Colella et al., 2024; Shibuya et al., 2022). However, these findings cannot currently be extrapolated to prion disease, as prion propagation is driven by self-templating PrP conversion rather than dysfunction of microglial populations. Therefore, microglial replacement remains a highly exploratory concept requiring direct validation in prion-specific models.

#### 3.6.2 Astrocytes as novel targets: pathological reactivity and emerging intervention strategies

Astrocytes represent another major glial population implicated in prion disease pathogenesis and should be considered alongside microglia as an important host-directed therapeutic target (Tahir et al., 2022; Kushwaha et al., 2023). Under physiological conditions, astrocytes maintain neuronal and synaptic homeostasis by regulating extracellular ion balance, glutamate uptake, metabolic support, blood–brain barrier integrity and trophic signaling (Murphy-Royal et al., 2023; Gradisnik and Velnar, 2023; Kim et al., 2026). During the prion disease, however, astrocytes undergo profound reactive transformation into neurotoxic subtype in response to PrP^Sc^ accumulation, Predominantly neuronal injury and microglia-derived inflammatory signals (Makarava et al., 2019; Bruno et al., 2023; Kushwaha et al., 2021; Baskakov, 2021; Hartmann et al., 2019).

One major mechanism by which astrocytes contribute to prion-induced neurotoxicity is through microglia–astrocyte cross-talk. Activated microglia can release inflammatory mediators such as IL-1α, TNF-α and C1q, which have been associated with the induction of neurotoxic reactive astrocyte phenotypes (Asano et al., 2020; Liddelow et al., 2017). Persistent exposure to these mediators may shift astrocytes away from homeostatic states and towards phenotypes characterized by reduced trophic and synaptic support (Liddelow et al., 2017). This interaction may contribute to a self-reinforcing glial response in which activated microglia promote astrocyte neurotoxicity, while dysfunctional astrocytes further impair neuronal resilience and amplify local inflammation. However, the extent to which this specific signaling pathway drives disease progression in human prion disease remains incompletely established. Some studies suggest that astrocytes may contribute to local spreading of pathological material, although the extent to which they function as direct reservoirs of prion replication remains context-dependent (Diedrich et al., 1991; Raeber et al., 1997; Krejciova et al., 2017). Even where astrocytes are not the primary source of PrP^Sc^ amplification, their loss of homeostatic function may indirectly accelerate disease by impairing glutamate clearance, increasing metabolic stress, disrupting synaptic support and promoting inflammatory amplification (Sofroniew and Vinters, 2010). These downstream effects may become particularly relevant during symptomatic prion disease.

The therapeutic relevance of astrocytes is supported by evidence that their reactive states can contribute actively to neurodegeneration. Makarava et al. (2019) proposed that reactive astrocytes in prion disease lose functions essential for neuronal support and acquire neurotoxic properties that may contribute to pathology (Makarava et al., 2019). Smith et al. (2020) further showed that the activation of the unfolded protein response in astrocytes induces a distinct reactive state that drives non-cell-autonomous neuronal degeneration. In their prion disease model, astrocytic PERK–eIF2*α* signaling altered astrocyte function, reduced synaptogenic support and contributed to neuronal loss, providing direct evidence that astrocyte stress pathways can influence disease progression (Smith et al., 2020). These findings identify astrocytic stress signaling and loss of homeostatic support as biologically plausible therapeutic targets. Potential approaches could include preserving astrocyte homeostatic functions, limiting maladaptive astrocyte reactivity, or modulating pathways such as UPR/PERK signaling (Smith et al., 2020). Nevertheless, the therapeutic evidence remain predominantly preclinical, and it is not yet clear whether astrocyte-directed intervention can reduce prion burden, prolong survival, or exhibit survival benefit when initiated after neurological symptoms emerge. Astrocyte reactivity is also functionally diverse, meaning that broad inhibition could inadvertently suppress protective responses.

Overall, astrocytes are active contributors to prion-associated synaptic dysfunction, inflammatory amplification, and neurodegeneration, rather than passive markers of reactive gliosis. Their translational value as therapeutic targets remains underdeveloped. Future studies should distinguish heterogenous astrocyte states, determine whether astrocyte-specific intervention modify survival or neurological decline in prion-infected animal models, and establish whether combined targeting of PrP propagation and glial dysfunction offers greater survival benefit than either approach alone.

### 3.7 Emerging preclinical therapeutic strategies

Although these approaches remain entirely preclinical, they provide mechanistic evidence that prion pathogenesis may be modified at multiple levels, including PrP conversion, substrate availability, protein aggregation, and downstream neurodegenerative pathways. One conversion-focused strategy is based on dominant-negative PrP biology (Westergard et al., 2011; Laffont-Proust et al., 2005; Vincent et al., 2001). Westergard et al. demonstrated that the naturally generated C-terminal PrP fragment C1 delayed prion disease progression and inhibited PrPSc formation, suggesting that promoting protective PrP processing or mimicking non-pathogenic PrP conformations may reduce conversion efficiency (Westergard et al., 2011; Laffont-Proust et al., 2005). Earlier studies further showed that altered PrP variants can interfere with prion replication in experimental models, supporting the broader concept that PrP conformational properties influence susceptibility to conversion (Vincent et al., 2001; Zulianello et al., 2000). A related example is the naturally occurring G127V PrP variant, identified in individuals exposed to kuru, which provides genetic evidence that specific PrP conformations can confer resistance to prion propagation (Asante et al., 2015). Asante et al. (2015) showed that G127V expression protected transgenic mice against multiple human prion isolates and exerted a dominant-negative effect when co-expressed with wild-type PrP. More recently, Gatdula et al. reported that the murine equivalent of G127V suppressed prion infection in cultured cell models, although these findings remain preliminary and have not yet been validated in animal therapeutic studies or clinical settings (Gatdula et al., 2026). Therefore, while dominant-negative PrP variants provide important mechanistic insights into prion conversion biology, their therapeutic translation remains highly speculative.

Other strategies aim to reduce downstream neuronal injury rather than directly blocking PrP^Sc^ formation. Mukherjee et al. reported that calcineurin inhibition with FK506 during the clinical phase of prion disease improved motor function, reduced synaptic and neuronal pathology, and prolonged survival in prion-infected mice (Mukherjee et al., 2010). This is important because treatment was given after clinical signs had emerged, suggesting that targeting neuronal stress pathways may remain beneficial even when disease is already established (Mukherjee et al., 2010). However, FK506 is an immunosuppressive drug, so its direct clinical application in prion disease would require cautious clinical evaluation in future.

Lithium-based treatment has also been explored as a host-directed therapeutic strategy (Relaño-Ginés et al., 2018; Song et al., 2017). Relaño-Ginés et al. showed that a low-dose lithium compound improved survival in prion-infected mice and reduced neuropathological changes, including vacuolation, astrogliosis and neuronal loss (Relaño-Ginés et al., 2018). This supports the idea that modulating neuroprotective, inflammatory or protein-clearance pathways may reduce downstream tissue damage. Nevertheless, lithium remains an experimental symptom-modifying treatment in prion disease, and its effects may depend on formulation, dose, timing and disease model (Relaño-Ginés et al., 2018).

A separate approach to treating prion disease involves the use of anti-aggregation small molecules (Wagner et al., 2013; Levin et al., 2014; European Clinical Trials Information Network, 2026). Anle138b, also known as Emrusolmin or TEV56286, is one of the best-studied examples showing promising therapeutic effect in animal models of neurodegeneration (Levin et al., 2014). Wagner et al. showed that anle138b was orally bioavailable, crossed the blood–brain barrier, reduced pathological PrP accumulation and prolonged survival in prion-infected mice (Wagner et al., 2013). Rather than lowering PrP^C^ expression, anle138b is designed to interfere with the formation and accumulation of toxic oligomeric protein assemblies (Levin et al., 2014; European Clinical Trials Information Network, 2026; Antonschmidt et al., 2022). This mechanism has also supported its development for synucleinopathies, where it is being investigated as a disease-modifying therapy targeting pathological α-synuclein aggregation (Antonschmidt et al., 2022). Early clinical studies in healthy volunteers indicated that it was generally well tolerated and achieved drug exposures above those associated with efficacy in animal models, while further trials are evaluating it in multiple system atrophy and Parkinson’s disease (Levin et al., 2022). However, these clinical programmes do not establish efficacy in human prion disease. Although its oral bioavailability, brain penetration and progression into clinical development strengthen the translational credibility of the compound, the therapeutic effect observed in prion disease remains preclinical (Wagner et al., 2013). Translation may also be complicated by prion strain diversity and by the possibility that aggregation inhibition alone may be insufficient once extensive gliosis, synaptic dysfunction and neuronal loss have developed.

More recently, Gate Bioscience has proposed “molecular gates” as a small-molecule PrP-lowering platform (Creutzfeldt-Jakob Disease International Support Alliance, 2026; Gate Bioscience, 2026). This strategy is designed to selectively eliminate PrP at its origin inside the cell by blocking its passage through the secretory pathway, thereby reducing the amount of PrP^C^ protein that reaches the cell surface and becomes available for conversion (Gate Bioscience, 2026). This approach remains attractive because it targets the upstream substrate required for prion propagation and may offer the practical advantages of small-molecule therapy. However, the Gate Bioscience prion programme remains at the preclinical stage and has not yet entered clinical application (Gate Bioscience, 2026).

## 4 Discussion and limitations

This review highlights a major transition in prion therapeutic development from screening of repurposed compounds towards mechanism-based approaches targeting fundamental processes involved in disease progression. While earlier compounds demonstrated anti-prion activity in experimental systems, their limited clinical success highlights the substantial challenges associated with modifying a rapidly progressive neurodegenerative disease driven by self-propagating protein misfolding. These challenges include delayed diagnosis, limited therapeutic windows, incomplete CNS target engagement, and the difficulty of translating findings from experimental models into human disease. The current therapeutic landscape suggests that approaches targeting the upstream mechanisms of prion propagation, particularly reduction of PrP availability, provide the strongest biological rationale for disease modification. However, clinical translation remains incomplete, and no intervention has yet demonstrated clear efficacy in human prion disease. Other approaches, including direct PrP immunotherapy and modulation of downstream pathological processes such as neuroinflammation and glial dysfunction, may provide complementary therapeutic opportunities but remain limited by uncertainties regarding efficacy, safety, and optimal timing of intervention. Importantly, emerging strategies such as genome editing, protective PrP variants, and other approaches should currently be regarded as exploratory concepts requiring further validation rather than established therapeutic candidates. The lack of clinical success to date also reflects broader challenges in prion therapeutic development rather than failure of individual therapeutic concepts alone. Human prion diseases are rare, rapidly progressive disorders, making clinical trial recruitment and evaluation of therapeutic benefit particularly challenging. Furthermore, most patients are diagnosed after substantial neuronal loss has occurred, when suppressing further prion propagation may be insufficient to restore neurological function. Therefore, future therapeutic development will depend not only on identifying effective molecular targets, but also on improving early diagnosis, developing reliable biomarkers for patient stratification and treatment monitoring, and establishing translational models that better predict human outcomes.

This review has several limitations. As a narrative scoping review, it does not provide a systematic assessment of all available evidence or formal risk-of-bias analysis. Furthermore, the therapeutic literature in prion disease spans diverse experimental systems, from cell-based studies to early clinical trials, which differ substantially in their ability to predict human efficacy. Findings derived from other neurodegenerative disease models, particularly for glial-directed or regenerative approaches, should therefore be interpreted cautiously due to differences in disease mechanisms and therapeutic requirements. Overall, future progress will depend on integrating biologically rational interventions with improved early diagnosis, validated biomarkers, and rigorous clinical evaluation. While no disease-modifying therapy is currently available, recent advances in PrP-targeting strategies provide a stronger foundation for translational development than previous approaches.

## 5 Concluding remarks

Prion disease remains one of the most therapeutically challenging neurodegenerative disorders, owing to its rapid clinical progression, diagnostic limitations, and the biological difficulty of targeting a self-propagating protein misfolding process within the central nervous system. Current evidence suggests that the most rational disease-modifying strategies are those that either reduce the availability of PrP^C^ or interfere with its pathogenic conversion, while downstream approaches targeting proteostatic dysfunction, neuroinflammation, and glial maladaptation may offer complementary benefit. Overall, the field is moving towards more mechanism-based and combinatorial therapeutic models, but substantial translational work remains necessary before these approaches can be considered clinically effective in human prion disease.

## Footnotes

1
https://pubmed.ncbi.nlm.nih.gov/

2
https://webofscience.zendesk.com/hc/en-us

3
https://scholar.google.com/
